# Tumor-Infiltrating Lymphocytes in Triple-Negative Breast Cancer: Prognostic Significance, Predictive Value, and Emerging Directions for Clinical Implementation

**DOI:** 10.3390/cancers18101588

**Published:** 2026-05-13

**Authors:** Panuch Eiamprapaporn, Cindy Venegas Mata, Raj Nandani, Thiti Susiriwatananont, Keith L. Knutson, Saranya Chumsri

**Affiliations:** 1 Department of Hematology and Medical Oncology, Mayo Clinic, Jacksonville, FL 32224, USA; susiriwatananont.thiti@mayo.edu (T.S.); chumsri.saranya@mayo.edu (S.C.); 2 Division of Medical Oncology, Department of Internal Medicine, Faculty of Medicine, Thammasat University, Pathum Thani 12120, Thailand; 3 Department of Pharmaceutical Sciences, South Dakota State University, Brookings, SD 57007, USA; cindy.venegasmata@jacks.sdstate.edu; 4 Department of Immunology, Mayo Clinic, Jacksonville, FL 32224, USA; nandani.raj@mayo.edu (R.N.); knutson.keith@mayo.edu (K.L.K.); 5 Department of Cancer Biology, Mayo Clinic, Jacksonville, FL 32224, USA; 6 Division of Medical Oncology, Department of Internal Medicine, Faculty of Medicine, Chulalongkorn University, Bangkok 10330, Thailand

**Keywords:** tumor-infiltrating lymphocytes, triple-negative breast cancer, immunotherapy, PD-L1, biomarkers, neoadjuvant therapy, pathologic complete response, tumor microenvironment, adoptive cell therapy

## Abstract

Triple-negative breast cancer (TNBC) represents a biologically aggressive subtype with limited therapeutic targets; however, tumor-infiltrating lymphocytes (TILs) have emerged as the most robust immune biomarker with compelling prognostic and predictive significance. This comprehensive review synthesizes current evidence on TIL assessment methodology; the immunobiological landscape of TNBC, including regulatory T cell populations and PD-L1 correlations; gene expression underpinnings of the tumor immune microenvironment; and systematic evidence from meta-analyses and clinical trials. Meta-analytic estimates demonstrate that high TIL levels are associated with improved overall survival (HR 0.58, 95% CI 0.48–0.71) and disease-free survival (HR 0.66, 95% CI 0.57–0.76), with each 10% TIL increment conferring incremental benefit. High TILs predict superior pathologic complete response to neoadjuvant chemotherapy (OR 2.14, 95% CI 1.43–3.19), with lymphocyte-predominant breast cancer achieving pCR rates exceeding 80% with pembrolizumab-based chemoimmunotherapy. Future directions include prospective TIL-guided treatment trials, artificial intelligence-enabled standardization, and emerging adoptive TIL cellular therapies for metastatic disease.

## Simple Summary

Triple-negative breast cancer is an aggressive type of breast cancer with few common treatment targets. Tumor-infiltrating lymphocytes (TILs) are immune cells found within tumors that reflect the body’s natural defense against cancer. Research has consistently shown that patients whose tumors contain more TILs have better outcomes and respond better to chemotherapy and immunotherapy. This review examines how TILs can guide treatment decisions for oncologists, surgeons, and pathologists working together to care for patients with triple-negative breast cancer. We synthesize evidence from systematic reviews and meta-analyses, demonstrating that high TIL levels predict improved survival and pathological complete response. Understanding TILs may help identify patients who need more intensive treatment and those who might safely receive less aggressive therapy, ultimately improving patient outcomes while reducing unnecessary side effects.

## 1. Introduction

Triple-negative breast cancer (TNBC) represents approximately 15–20% of all breast cancers and is characterized by the absence of estrogen receptor, progesterone receptor, and HER2 expression [1,2]. This subtype is associated with aggressive clinical behavior, early recurrence, and limited targeted therapeutic options compared with other breast cancer subtypes. Despite these challenges, TNBC is increasingly recognized as one of the most immunogenic forms of breast cancer, characterized by high levels of genomic instability, increased tumor mutational burden, and a prominent immune-infiltrated tumor microenvironment [3,4,5].

Among the components of the immune microenvironment, tumor-infiltrating lymphocytes (TILs) have emerged as one of the most reproducible and clinically relevant biomarkers in TNBC [6,7]. TILs reflect the host immune response against tumor cells and consist primarily of cytotoxic CD8+ T cells, helper CD4+ T cells, regulatory T cells, B cells, and natural killer (NK) cells that collectively influence tumor progression and therapeutic response [8]. The presence of abundant TILs indicates an active antitumor immune response, often associated with increased recognition of tumor neoantigens and enhanced immune surveillance [9,10].

Accumulating evidence has demonstrated that elevated TIL levels are strongly associated with improved clinical outcomes in TNBC. Numerous retrospective analyses and prospective clinical trials have shown that higher stromal TIL levels correlate with improved disease-free survival, overall survival, and an increased likelihood of achieving a pathologic complete response following neoadjuvant chemotherapy [11,12,13,14]. The landmark JAMA 2024 analysis by Leon-Ferre et al., representing the International Immuno-Oncology Biomarker Working Group, pooled individual patient-level data from 1966 participants with early-stage triple-negative breast cancer (TNBC) who were not treated with adjuvant or neoadjuvant chemotherapy across 13 international centers, demonstrating that each 10% increment in TIL abundance was independently associated with improved overall survival (HR 0.88; 95% CI 0.85–0.91) [1]. In addition to their prognostic value, TILs also appear to have predictive significance for response to immune checkpoint inhibitors, particularly therapies targeting the PD-1/PD-L1 pathway [15,16,17]. These findings highlight the importance of the tumor immune microenvironment as a key determinant of therapeutic sensitivity in TNBC.

From a biological perspective, the recruitment and functional activity of TILs are governed by complex interactions between tumor cells, antigen-presenting cells, and immune regulatory pathways. Tumor neoantigen presentation, dendritic cell activation, interferon signaling, and chemokine-mediated chemotaxis contribute to the infiltration of cytotoxic lymphocytes into the tumor microenvironment. Conversely, immunosuppressive cells, such as regulatory T cells and myeloid-derived suppressor cells, and immune checkpoint signaling can limit effective antitumor immunity and facilitate immune escape [18,19]. The balance between immune activation and immune evasion within the tumor microenvironment ultimately determines clinical outcomes and therapeutic responsiveness.

Given the growing clinical relevance of immunotherapy in TNBC, there is increasing interest in integrating TIL assessment into precision oncology frameworks. Standardized evaluation of stromal TILs has been proposed by the International Immuno-Oncology Biomarker Working Group, providing a reproducible and cost-effective approach for quantifying immune infiltration in routine histopathology [5,20]. However, important questions remain regarding the biological mechanisms driving TIL heterogeneity, the optimal integration of TIL assessment with other immune biomarkers such as PD-L1 expression and gene expression signatures, and the clinical utility of TIL-guided treatment strategies for de-escalation in lymphocyte-predominant tumors or escalation in immune-desert tumors.

**Figure 1:**
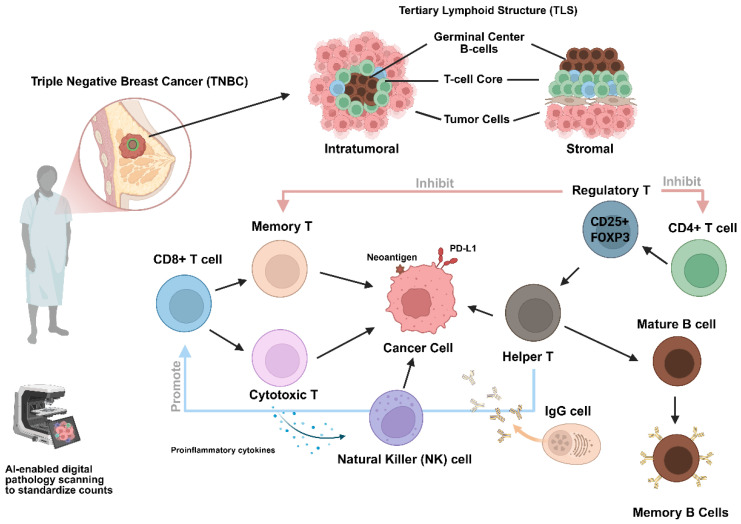
Evolving landscape of immuno-oncology. Created in BioRender. Venegas Mata, C. (2026) https://BioRender.com/66ekr5g.

In this review, we synthesize current evidence on the biological mechanisms, prognostic significance, and predictive value of tumor-infiltrating lymphocytes in triple-negative breast cancer. We examine the immunoregulatory landscape, including regulatory T cell biology, B cell and NK cell contributions, tertiary lymphoid structures, and spatial immune architecture; explore gene expression correlates of TIL infiltration; present systematic evidence from meta-analyses and clinical trials; and discuss ongoing clinical trials incorporating TIL assessment for treatment personalization. We also address emerging adoptive TIL cellular therapies and the potential role of artificial intelligence in standardizing TIL assessment within the evolving landscape of immuno-oncology (Figure 1).

## 2. Immune Regulation in Context: Integrating Treg Biology, Checkpoint Expression, and Spatial Architecture in TNBC

### 2.1. Tumor Immune Escape and the Biological Basis of TIL-Associated Prognosis

Although increased leukocyte infiltration is generally associated with improved clinical outcomes, this correlation likely reflects the pressure exerted by the immune system on tumor cells rather than a simple beneficial effect of immune cell presence [21]. Tumors develop under continuous immune surveillance, and those that remain detectable often represent lesions in which immune-mediated elimination is incomplete but ongoing [22]. In contrast, non-infiltrated or “immune-cold” tumors are increasingly recognized as tumors that have successfully adopted immune escape strategies, allowing them to avoid immune recognition altogether [23].

Multiple mechanisms contribute to tumor immune evasion. These include defects in antigen processing and presentation, such as loss of HLA class I molecules, which impair T cell recognition [24]. Tumors also actively suppress antitumor immunity through the expression of immune checkpoint ligands (e.g., PD-L1), secretion of immunosuppressive cytokines such as TGF-β, recruitment of regulatory T cells and myeloid-derived suppressor cells, and metabolic reprogramming within the tumor microenvironment. In addition, structural and stromal barriers, including aberrant vasculature and dense extracellular matrix, can physically exclude immune cells from the tumor core, resulting in immune-excluded phenotypes [25].

From an evolutionary perspective, these mechanisms are selected through immunoediting, whereby immune pressure eliminates highly immunogenic tumor clones while favoring the outgrowth of variants capable of immune escape. Consequently, tumors with minimal immune infiltration likely represent advanced stages of immune adaptation rather than a lack of immunogenicity. This framework provides a biological explanation for the association between leukocyte-rich tumors and better prognosis and supports the interpretation that immune infiltration is a marker of ongoing, albeit imperfect, immune control.

### 2.2. Regulatory T Cells as a Functional Component of the TNBC Immune Microenvironment

The immune microenvironment of TNBC comprises diverse cell populations with opposing functional roles. Cytotoxic CD8+ T lymphocytes mediate antitumor immunity, while immunoregulatory populations are equally critical in shaping immune equilibrium and therapeutic responsiveness. Among these, regulatory T cells (Tregs) constitute a key immunosuppressive compartment that modulates effector T cell activation, immune tolerance, and inflammatory balance within tumors [18,26].

Tregs are classically defined by the CD4+CD25+ phenotype, with FOXP3 (forkhead box P3) expression serving as the master transcriptional regulator responsible for their development, stability, and suppressive function [27,28]. FOXP3 governs multiple immunoregulatory pathways, including inhibition of effector T cell proliferation, cytokine modulation, and maintenance of peripheral immune tolerance. Consequently, FOXP3 expression has been widely used as a surrogate marker for Tregs in tumor-infiltrating lymphocyte (TIL) studies.

### 2.3. Functional Heterogeneity of CD4+CD25+ TILs and the FOXP3 Paradox

The clinical significance of FOXP3+ TILs in TNBC is context-dependent. The meta-analysis by Shou et al. demonstrated that higher FOXP3+ TIL levels in breast cancer were associated with worse overall survival [23]. However, this relationship is not straightforward: FOXP3+ cells often coexist with dense cytotoxic immune infiltration in immunologically ‘hot’ tumors, and their presence may reflect active immune engagement rather than purely immunosuppressive activity. The ratio of effector to regulatory T cells—rather than Treg density alone—may more accurately predict clinical outcomes. This observation reflects a fundamental principle of tumor immunology: immune cell subsets do not function in isolation.

Importantly, CD25 expression is not exclusive to regulatory T cells. Activated effector T cells responding to tumor antigens also transiently express CD25, complicating the interpretation of CD4+CD25+ populations. Cai et al. demonstrated that FOXP3+ Tregs represent only a minority of CD4+CD25+ TILs in TNBC and exhibit distinct expression patterns of immunoregulatory molecules, including CTLA-4, LAG-3, and TGF-β, compared with FOXP3-negative counterparts [29]. This distinction carries important therapeutic implications for strategies targeting CD25 without accounting for FOXP3 expression [7,27,28].

### 2.4. Spatial Immune Architecture and Clinical Relevance: Insights from the IBCSG 22-00 Trial

Beyond immune cell composition, the spatial organization of immune infiltrates within the tumor microenvironment has emerged as a critical determinant of clinical outcome [30]. The International Breast Cancer Study Group (IBCSG) Trial 22-00 provides one of the most comprehensive spatial immune analyses conducted in high-risk TNBC. Using multiplex high-throughput immunofluorescence, Rusakiewicz et al. characterized immune cell populations, including CD3+, CD4+, CD8+, and FOXP3+ T cells, across stromal and intra-epithelial compartments. Crucially, the analysis revealed that immune spatial distribution—not immune density alone—determines therapeutic impact. Patients with T cell-excluded tumors experienced inferior outcomes when treated with cyclophosphamide–methotrexate maintenance, suggesting that baseline immune architecture can predict not only lack of benefit but potential treatment harm [31].

### 2.5. PD-L1 Expression as a Complementary Immune Dimension

Programmed death-ligand 1 (PD-L1) expression represents an additional layer of immune regulation, reflecting adaptive immune resistance mechanisms within the tumor microenvironment. Across multiple studies, PD-L1 expression in TNBC has shown a moderate positive correlation with both stromal and intratumoral TILs [32,33,34], reinforcing its association with immune-inflamed tumors.

However, PD-L1 expression and TIL abundance capture distinct yet complementary aspects of tumor immunogenicity. While TILs reflect immune infiltration and host immune engagement, PD-L1 expression reflects dynamic immune suppression in response to inflammatory signaling. Salgado et al. proposed a combined PD-L1 and TIL assessment framework to optimize patient selection for immune checkpoint inhibitor therapy [20,35].

### 2.6. Conceptual Integration

**Figure 2:**
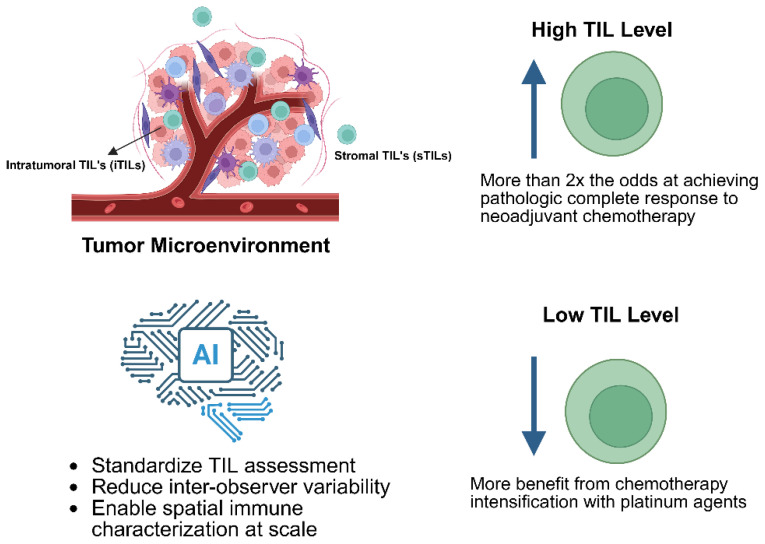
Immune regulation in TNBC. Created in BioRender. Venegas Mata, C. (2026) https://BioRender.com/neullok.

Taken together, regulatory T cells, spatial immune architecture, and PD-L1 expression collectively illustrate that immune regulation in TNBC is multidimensional. Effective interpretation of immune biomarkers requires integration of cellular identity, functional heterogeneity, spatial organization, and immune checkpoint activity. This framework provides the biological foundation for subsequent sections exploring gene expression correlates, systematic evidence, and biomarker-guided therapeutic strategies (Figure 2).

## 3. Molecular and Transcriptomic Correlates of TIL Infiltration in TNBC

### 3.1. Molecular Subtypes of TNBC and Immune Signatures

TNBC is molecularly heterogeneous, with gene expression profiling revealing distinct subtypes with differential immune characteristics and therapeutic sensitivities [36,37]. The original TNBC type classification identified four molecular subtypes, including an immunomodulatory (IM) subtype characterized by elevated expression of immune-related genes. Subsequent refinement to TNBC type-4 recognized that transcripts in the IM subtype were contributed substantially from tumor-infiltrating lymphocytes rather than tumor cells themselves, resulting in four tumor-specific subtypes: basal-like 1 (BL1), basal-like 2 (BL2), mesenchymal (M), and luminal androgen receptor (LAR) [38].

### 3.2. Gene Expression Correlates of TIL Infiltration

Han et al. conducted a comprehensive characterization of TIL composition in TNBC using NanoString nCounter PanCancer Immune Profiling, revealing significant upregulation of cytotoxic T lymphocyte (CTL)- and natural killer (NK) cell-associated genes, including GNLY, KLRC2, and GZMB, in TIL-high tumors [30]. The development of a CTL-NK score demonstrated independent prognostic value for disease-free survival.

Jovanović et al. performed integrative multiomic profiling of TNBC using the NanoString BC360 platform combined with targeted DNA sequencing [39]. This approach quantified TILs through H&E staining while simultaneously assessing retinoblastoma, androgen receptor, and PD-L1 proteins through immunohistochemistry and performing comprehensive transcriptomic profiling. Key findings demonstrated that PD-L1 expression—whether assessed on immune cells or tumor cells—identified tumors with similar underlying biology characterized by elevated immune activation signatures. Furthermore, high stromal TIL levels correlated strongly with immune gene expression modules, validating the biological relevance of histopathologic TIL assessment as a surrogate for transcriptomic immune activity.

### 3.3. Spatial Immune Microlandscapes

Beyond bulk immune infiltration, the spatial organization of immune cells within the tumor microenvironment carries independent prognostic significance. Carter et al. identified distinct spatial immune microlandscapes in TNBC that were independently associated with outcomes [40]. Specifically, tumors characterized by direct immune cell contact with tumor nests (inflamed phenotype) demonstrated superior prognosis compared to tumors with immune cells confined to the stromal compartment (excluded phenotype), even when overall TIL density was comparable. The distinction between immune-inflamed, immune-excluded, and immune-desert phenotypes has emerged as clinically relevant in predicting responses to immune checkpoint inhibitors [41,42].

## 4. Systematic Evidence: TILs for Prognosis and Prediction

### 4.1. Prognostic Value of TILs in Early-Stage TNBC

The most comprehensive evidence for the prognostic value of TILs comes from the systematic review and meta-analysis by Gao et al. (2020), which synthesized data from 37 studies [43]. When comparing high versus low TIL levels, patients with high TILs exhibited significantly improved overall survival (OS; HR 0.58, 95% CI 0.48–0.71, *p* < 0.001) and disease-free survival (DFS; HR 0.66, 95% CI 0.57–0.76, *p* < 0.001). When analyzed as a continuous variable, each 10% increase in TIL percentage was associated with OS improvement (HR 0.90, 95% CI 0.87–0.93) and DFS improvement (HR 0.92, 95% CI 0.90–0.95). Key clinical trials incorporating TIL assessment in TNBC are summarized in Table 1.

### 4.2. TIL Subsets and Phenotype-Specific Prognostic Value

The meta-analysis by Gao et al. also examined specific TIL subpopulations identified by immunohistochemistry [43]. CD4+ T cells demonstrated particularly strong prognostic associations (OS HR 0.49, 95% CI 0.32–0.76; DFS HR 0.54, 95% CI 0.36–0.80). CD8+ cytotoxic T cells showed significant associations with DFS (HR 0.55, 95% CI 0.38–0.81) but not OS (HR 0.70, 95% CI 0.46–1.06). FOXP3+ regulatory T cells demonstrated significant DFS associations (HR 0.50, 95% CI 0.33–0.75) with wide confidence intervals for OS.

### 4.3. Predictive Value for Neoadjuvant Chemotherapy

TILs have demonstrated strong predictive value for pathologic complete response (pCR) following neoadjuvant chemotherapy. The meta-analysis by Gao et al. demonstrated that high versus low TILs yielded an odds ratio of 2.14 (95% CI 1.43–3.19) for pCR [43]. The Neo-Real study by Barroso-Sousa et al. (2024) investigated optimal TIL cutoff values in 128 TNBC patients treated with neoadjuvant pembrolizumab plus chemotherapy [47]. At the ≥50% cutpoint (lymphocyte-predominant breast cancer), pCR rates reached 87.5% versus 55.4% for TILs < 50%. At ≥30%, pCR was 78.8% versus 52.9%; at ≥10%, pCR was 69.0% versus 38.5%. Proposed TIL cutpoints and their clinical implications are summarized in Table 2.

### 4.4. Predictive Value for Immunotherapy Response

Multiple studies have evaluated TILs as predictive biomarkers for response to PD-1/PD-L1 checkpoint inhibitors. The NeoPACT trial enrolled 110 stage I–III TNBC patients treated with carboplatin, docetaxel, and pembrolizumab [49]. sTILs as a continuous variable were predictive of pCR (OR 1.022, 95% CI 1.009–1.035, *p* = 0.001). Wood et al. (2024) analyzed 76 patients treated with the KEYNOTE-522 regimen, with pre-treatment TILs significantly associated with pCR (mean TIL 28.5% in pCR vs. 9.73% in residual disease, *p* = 0.014) [50].

Sharma et al. (2023) analyzed the TNBC-DX gene signature, finding that the 14-gene B cell signature was significantly associated with improved pCR (OR 1.105, 95% CI 1.019–1.197), with pCR rates of 71% in IGG-high versus 44% in IGG-low groups (OR 3.152) [48].

### 4.5. Real-World Evidence: Differential Treatment Benefit by TIL Status

The retrospective study by Bae et al. (2024) analyzed 450 TNBC patients treated with neoadjuvant chemotherapy [46]. In **lymphocyte-predominant breast cancer (LPBC)** patients (≥50% sTILs), pCR rates were 83.3% with pembrolizumab plus carboplatin. In contrast, non-LPBC patients showed pCR rates of 61.2% with pembrolizumab-based therapy (no significant difference from carboplatin alone). These findings suggest differential treatment benefit: LPBC patients derive substantial benefit from immunotherapy, while non-LPBC patients benefit primarily from platinum-based chemotherapy intensification.

### 4.6. TILs in Metastatic TNBC

Evidence for TILs in metastatic TNBC is emerging. Heeke et al. (2021) reported that a higher density of CD8+ cytotoxic TILs correlated with improved PFS and OS in IMpassion130 [16,51,52]. Geurts and Kok emphasized that biomarkers capturing immunogenicity—including TILs, CD8 T cell levels, and interferon-gamma signatures—could support treatment selection in metastatic TNBC [51].

### 4.7. TILs and Immune-Related Adverse Events

An emerging area of investigation is the relationship between baseline immune characteristics and immune-related adverse events (irAEs) following checkpoint inhibitor therapy [53]. Preliminary data suggest that patients with higher baseline TILs or specific immune gene signatures may be at increased risk for irAEs, although this relationship requires further characterization in TNBC-specific cohorts [54].

## 5. Ongoing Clinical Trials Incorporating TIL Assessment

### 5.1. TILs as Predictive Biomarkers for Treatment Personalization

Multiple ongoing clinical trials are prospectively incorporating TIL assessment as stratification factors or eligibility criteria to guide treatment intensity [44,55,56]. These trials represent a paradigm shift toward biomarker-driven treatment personalization in TNBC (Table 3).

De-escalation trials are investigating whether high TIL levels can identify early-stage TNBC patients who may safely omit components of standard neoadjuvant chemoimmunotherapy. The ETNA Trial (NCT06078384) investigates a chemotherapy de-escalation strategy for early-stage TNBC, where patients with high TIL levels (≥30% or ≥50%, depending on age) may receive reduced chemotherapy or standard surveillance to avoid overtreatment [57]. The NeoTRACT Trial is an adaptive phase II trial that assigns treatment based on baseline sTILs: patients with >30% sTILs receive only 4 cycles of therapy, while those with lower levels receive more intensive regimens [58]. Similarly, the TIL-Driven De-escalation trial (NCT07074106) focuses on Stage I–II TNBC, using TIL levels ≥ 50% to guide a de-escalated neoadjuvant chemotherapy regimen [59].

The BELLINI Trial represents one of the first trials to select patients according to TILs to orient treatment, specifically testing a ‘window of opportunity’ approach with immunotherapy (nivolumab ± ipilimumab) in TIL-selected patients [60]. The NeoPACT and KEYNOTE-522 trials have evaluated TILs as secondary endpoints to assess their predictive value for pathologic complete response [17,44,45].

Escalation trials are evaluating whether low-TIL tumors—presumably less immunogenic—benefit from strategies to enhance immune infiltration before or concurrent with checkpoint inhibitor therapy. These approaches include radiation therapy to promote immunogenic cell death, targeted agents that modulate the tumor microenvironment, and novel combination strategies.

### 5.2. Adoptive TIL Therapy: Cellular Immunotherapy Approaches in TNBC

Tumor-infiltrating lymphocytes are a polyclonal, tumor-specific population derived from a patient’s own tumor microenvironment. Expanded ex vivo and reinfused, they can recognize and kill tumor cells more broadly than single-antigen therapies like CAR-T cells, especially in cancers with high mutational burden like TNBC. Several clinical trials are now evaluating adoptive TIL therapy in advanced or metastatic TNBC.

NUMARZU-001 (NCT05451784) is a Phase I/II trial evaluating PD1-positive TILs in advanced/metastatic TNBC [61]. The trial employs molecular pre-screening of archival tumor tissue for PD-1 mRNA expression to select patients likely to yield tumor-reactive TILs. This approach is grounded in translational immunology evidence that PD-1 upregulation marks exhausted but potentially reactive T cells that may regain cytotoxic function upon ex vivo expansion and reinfusion. Endpoints include safety (grade 3–5 adverse events) and clinical activity (overall response rate, progression-free survival at 6 months).

GC101 (Shanghai Juncell Therapeutics) is an autologous TIL product designed to be IL-2-independent with low-intensity preconditioning, potentially improving tolerability compared to conventional TIL protocols that require high-dose IL-2 post-infusion. The study suggests a manageable safety profile across solid tumors, including TNBC, representing an important evolution in TIL therapy procedures [62].

LN-145 (NCT03449108) is a completed Phase II study of autologous TILs in metastatic TNBC conducted by Yale University and Iovance Biotherapeutics [63]. Patients received non-myeloablative lymphodepletion followed by TIL infusion and up to six doses of IL-2 support. The trial enrolled 6 patients, reflecting the resource-intensive nature of adoptive TIL therapy in refractory populations. Detailed efficacy results for the TNBC cohort have not been publicly reported; however, the completed trial provides proof-of-concept for TIL manufacturing and delivery in this setting. LN-145 represents one of the few adoptive TIL therapies tested specifically in metastatic TNBC, and the favorable efficacy profile observed with lifileucel in melanoma and NSCLC supports further exploration of cellular therapies in this aggressive subtype.

These approaches represent a shift from conventional cytotoxic therapies and checkpoint blockade alone toward personalized cellular immunotherapy for TNBC—a subtype that has historically had limited targeted therapy options. Continued evaluation in controlled trials, including biomarker-driven designs, will be critical to determine clinical benefit and optimize patient selection.

## 6. Future Directions and Clinical Implementation

### 6.1. Standardization and Quality Assurance

The transition of TILs from research biomarker to routine clinical application requires standardization, quality assurance, and consensus on reporting practices [20]. The International Immuno-Oncology Biomarker Working Group has published recommendations for TIL assessment methodology, including standardized scoring guidelines available at www.tilsinbreastcancer.org [64]. Key elements include evaluation on H&E-stained sections, scoring stromal TILs as a percentage of stromal area within invasive tumor borders, and reporting as a continuous variable.

### 6.2. Artificial Intelligence in TIL Assessment

Artificial intelligence (AI)-enabled digital pathology represents a pivotal advancement for standardizing TIL assessment in clinical practice [65]. Deep learning-based computational approaches enable automated, objective, and scalable quantification of immune infiltration from routine H&E-stained whole-slide images.

Rasic et al. (2025) validated an AI-based TIL assessment tool in 518 patients, demonstrating a high correlation with pathologist scores (Spearman r = 0.61–0.77) and significant associations with DFS and OS in TNBC [66]. Schirris et al. (2025) developed the label-efficient computational TIL assessment model (ECTIL), validated in 2340 patients, showing that every 10% increase in ECTIL scores was associated with improved OS (HR 0.86) [67]. Vidal et al. (2024) addressed AI algorithm interchangeability for TIL scoring, demonstrating analytical and clinical validity [68].

### 6.3. Clinically Actionable Cutpoints

A critical unresolved question is the definition of clinically actionable TIL cutpoints for treatment decision-making. The JAMA 2024 pooled analysis demonstrated that 5-year distant recurrence-free survival for stage I TNBC was 94% for patients with ≥50% TILs compared to 78% for those with <30% TILs [1]. A ≥50% TIL threshold identifies a population with an exceptionally favorable prognosis that may be candidates for treatment de-escalation.

### 6.4. Multidisciplinary Integration

The clinical utility of TILs depends on effective multidisciplinary communication among pathologists, medical oncologists, and breast surgeons [6,69]. Tumor board discussions should incorporate TIL results alongside PD-L1 status, molecular subtyping, and clinical staging. The combination of high TILs, PD-L1 positivity, and favorable tumor characteristics may identify patients for de-escalation trials, while the converse pattern may prompt consideration of escalation strategies.

### 6.5. Research Priorities

Key research priorities include prospective clinical trials with pre-specified TIL-based stratification for treatment de-escalation in LPBC and treatment intensification in low-TIL tumors. Development of combination biomarker models integrating TILs with PD-L1 expression, genomic signatures, and spatial analysis represents an important avenue. Studies evaluating concordance between primary and metastatic TIL levels would also inform whether primary tumor TIL assessment can guide treatment decisions in advanced disease. Additionally, continued development of adoptive TIL cellular therapies may provide new treatment options for patients with refractory disease.

## 7. Conclusions

Tumor-infiltrating lymphocytes represent the most extensively validated immune biomarker in breast cancer, with particularly robust prognostic and predictive significance in TNBC. The biological foundation of TIL assessment—reflecting the endogenous anti-tumor immune response—positions TILs as a physiologically meaningful biomarker that integrates tumor immunogenicity, host immune competence, and tumor–stroma interactions.

Beyond prognosis, TILs demonstrate predictive value for treatment response. High TIL levels predict more than double the odds of pCR to neoadjuvant chemotherapy. In the immunotherapy era, lymphocyte-predominant breast cancer patients achieve pCR rates exceeding 80% with pembrolizumab-based chemoimmunotherapy, while non-LPBC patients appear to benefit primarily from chemotherapy intensification.

The clinical trial landscape is rapidly evolving, with multiple de-escalation and escalation trials now incorporating TIL assessment for treatment personalization. Emerging adoptive TIL cellular therapies offer potential new options for patients with metastatic disease, leveraging the tumor-specific reactivity of ex vivo expanded lymphocytes.

Artificial intelligence offers the potential to standardize TIL assessment, reduce inter-observer variability, and enable spatial immune characterization at scale. TILs occupy a unique position in TNBC research and practice: biologically grounded, clinically validated, technically accessible, and universally affordable. The next phase of progress depends not on discovering new biomarkers but on fully leveraging those already proven through prospective trials, standardized assessment, and multidisciplinary implementation.

## Figures and Tables

**Table 1:** Key clinical trials incorporating TIL assessment in TNBC.

Trial	Phase	Patient Number	Drug	Setting	TIL Finding	Key Outcome	Reference
JAMA 2024 Pooled	Meta analysis	1966	No adjuvant or neoadjuvant chemotherapy	Early TNBC	10% TIL increment	OS HR 0.88	[1]
Gao Meta-Analysis	Meta-analysis	128	Neoadjuvant pembrolizumab and chemotherapy	Early TNBC	High vs. low TILs	OS HR 0.58; pCR OR 2.14	[43]
KEYNOTE-522	III	76	Neoadjuvant pembrolizumab with chemotherapy pre-surgery, followed by adjuvant pembrolizumab post-surgery for high-risk early-stage TNBC	Neoadjuvant	Benefit regardless PD-L1	pCR 64.8% vs. 51.2%	[17,44]
NeoPACT	I–III	110	Carboplatin, docetaxel, and pembrolizumab	Neoadjuvant	sTILs predictor	pCR OR 1.02 per 1%	[45]
Bae Real-World	“early”	450	247 non-carboplatin, 120 carboplatin, and 83 pembrolizumab	Neoadjuvant	LPBC differential	LPBC pCR 83.3%	[46]
IBCSG 22-00	III	1086	CM or no CM	Adjuvant	Spatial critical	T-excluded poor	[31]

TIL: tumor-infiltrating lymphocytes; OS: overall survival; HR: hazard ratio; pCR: pathologic complete response; OR: odds ratio; LPBC: lymphocyte-predominant breast cancer; CM: cyclophosphamide and methotrexate.

**Table 2:** Proposed TIL cutpoints and clinical implications.

TIL Level	Prognostic Implication	Potential Clinical Action
≥50% (LPBC)	Excellent prognosis; pCR 83–87%	De-escalation trials candidate
≥30% (High)	Favorable; pCR ~79%	Standard chemoimmunotherapy
≥10% (Intermediate)	Moderate; pCR ~69%	Standard therapy
<10% (Low)	Higher risk; pCR ~39%	Intensification; escalation trials

TIL: tumor-infiltrating lymphocytes; LPBC: lymphocyte-predominant breast cancer; pCR: pathologic complete response. Cutpoints derived from pooled analyses [1,46,48] and real-world data [44].

**Table 3:** Ongoing and completed TIL-based clinical trials in TNBC.

Trial	Phase	Setting	TIL Strategy	Key Design Feature	Study Number
ETNA	II	Early TNBC	Predictive	De-escalation if TIL ≥ 30–50%	NCT06078384
NeoTRACT	II	Neoadjuvant	Predictive	Adaptive dosing by sTILs	NCT05645380
TIL De-escalation	II	Stage I–II	Predictive	De-escalation if TIL ≥ 50%	NCT07074106
BELLINI	II	Window	Predictive	ICI selection by TILs	EGAS50000000568
LN-145	II	Metastatic	Adoptive	Autologous TIL + IL-2	NCT03449108
NUMARZU-001	I/II	Metastatic	Adoptive	PD1+ TIL selection	NCT05451784

TIL: tumor-infiltrating lymphocytes; ICI: immune checkpoint inhibitor; IL-2: interleukin-2; NCT: ClinicalTrials.gov identifier.

## Data Availability

Not applicable.
